# Do cavies talk? The effect of anthropomorphic picture books on children's knowledge about animals

**DOI:** 10.3389/fpsyg.2014.00283

**Published:** 2014-04-10

**Authors:** Patricia A. Ganea, Caitlin F. Canfield, Kadria Simons-Ghafari, Tommy Chou

**Affiliations:** ^1^Applied Psychology and Human Development, University of TorontoToronto, ON, Canada; ^2^Department of Psychological and Brain Sciences, Boston UniversityBoston, MA, USA; ^3^Department of Psychology, Florida International UniversityMiami, FL, USA

**Keywords:** picture books, preschoolers, learning, animals, anthropomorphism

## Abstract

Many books for young children present animals in fantastical and unrealistic ways, such as wearing clothes, talking and engaging in human-like activities. This research examined whether anthropomorphism in children's books affects children's learning and conceptions of animals, by specifically assessing the impact of depictions (a bird wearing clothes and reading a book) and language (bird described as talking and as having human intentions). In Study 1, 3-, 4-, and 5-year-old children saw picture books featuring realistic drawings of a novel animal. Half of the children also heard factual, realistic language, while the other half heard anthropomorphized language. In Study 2, we replicated the first study using anthropomorphic illustrations of real animals. The results show that the language used to describe animals in books has an effect on children's tendency to attribute human-like traits to animals, and that anthropomorphic storybooks affect younger children's learning of novel facts about animals. These results indicate that anthropomorphized animals in books may not only lead to less learning but also influence children's conceptual knowledge of animals.

## Introduction

For most young children, picture-book interaction is an important part of daily life. A growing body of research examines children's learning and transfer of information encountered in picture books to the real world (Simcock and DeLoache, 2006, 2008; Simcock and Dooley, 2007; Ganea et al., 2008, 2009, 2011; Tare et al., 2010; Simcock et al., 2011; Walker et al., 2012b; Khu et al., 2014; Keates et al., in press). This research has established that some elements of picture books (e.g., pictorial realism, manipulative features) impact children's ability to learn and transfer information from books. For example, across several studies using different methods it was found that more iconic pictures lead to better learning and transfer from picture books in young children than less iconic pictures do (Simcock and DeLoache, 2006; Ganea et al., 2008, 2009; Tare et al., 2010). In other words, the higher the level of similarity between the picture and the referent, the easier it is for children to transfer information between the two. Manipulative books are books that contain elements that a child could physically interact with, such as flaps to lift, textures to feel, tabs to pull, and so on. These elements are thought to make books engaging to young children, yet the research so far suggests that manipulative books may not be advantageous for learning. Studies that compared children's learning of content from simple, traditional books vs. manipulative books have found negative effects of manipulative elements on children's learning (Tare et al., 2010; Chiong and DeLoache, 2012).

Another important feature of picture books that can potentially impact children's learning has to do with the nature of the relation between the depiction and the referent, that is whether the depictions in picture books portray real entities in a realistic or fantastical manner. Many picture books for infants and young children depict reality in a distorted way. Human consciousness, knowledge, abilities, purpose, and intentions are often attributed to animal characters (e.g., seals solve mysteries, cats build houses and mice drive cars) and even to inanimate objects (e.g., lamps have faces and dance the tango, trains strive against all odds to achieve impossible goals). Fantasy elements are often employed even in books designed to convey serious information about the real world, including books with a focus on scientific knowledge. One question is whether the use of anthropomorphic elements in books might be counterproductive for learning (Ganea et al., 2011). Would the use of anthropomorphism to describe animals in picture books affect children's conceptual knowledge of real animals? In other words, does seeing animals talk and engage in human-like activities in children's picture books affect children's understanding of the biological and psychological properties of real non-human animals?

Urban 4- and 5-year-old children tend to use an anthropocentric model when reasoning about the biological world (Carey, 1985; Springer and Keil, 1989; Waxman and Medin, 2007). When asked to explain the behavior of non-human animals, preschool children take the human as the prototype and transfer properties from humans to other animals broadly. However, if a novel property is introduced in relation to a non-human animal (e.g., a dog), urban 4- and 5-year-olds are less likely to project it to humans. Recent work on children's biological reasoning has shown that, compared to urban 5-year-old children, the anthropocentric perspective on the biological world is not as predominant in urban 3-year-old children (Herrmann et al., 2010) and in children in rural communities who have more direct contact with animals (Waxman and Medin, 2007).

What explains these cultural and developmental differences regarding children's anthropocentric view of non-human animals? One possibility is that children growing up in different cultural settings (e.g., rural vs. urban) have different opportunities for informal learning about animals. In urban settings, children's direct exposure to animals is often limited and the books and media for young children depict animals in an anthropomorphic way (Inagaki, 1990; Rosengren et al., 1991). Also, with increased exposure with age to media that anthropomorphizes animals, children's view of animals may become more human-centered. Presenting animals to children in ways that are similar to how humans act and behave is likely to be counter-productive for learning scientifically accurate information about the biological world and to influence children's view of the biological world (Ganea et al., 2011).

Recent research on children's learning and transfer of knowledge from picture books has shown that children are more likely to transfer information from picture books when the information is presented in a story context “close” to the real world than in a context that is more dissimilar to the real world (Walker et al., 2012a). If children learned about a cause-effect relation from a story that depicted a boy who participated in realistic activities (e.g., having a picnic) they were more likely to use that causal information to explain a real life event than when they were exposed to a story about a boy who had a more fantastical experience (e.g., talking to a tree). This study indicates that the “proximity” of the story context to the real world affects the extent to which children transfer content from the book to reality (Walker et al., 2012a). In other words, children are sensitive to whether the structure of the story world resembles the structure of the real world, and their learning is disrupted if content information is portrayed in a “far” fantastical context. Thus, in terms of transfer of content knowledge, the existing evidence indicates that children are less likely to transfer content information from fantastical books to the real world compared to realistic books (Richert et al., 2009; Richert and Smith, 2011; Walker et al., 2012a). Adding fantasy elements to books that are supposed to teach children novel facts about animals could make it less likely that children will learn and transfer those facts.

In addition, adding fantastical elements in picture books could lead children to adopt an anthropocentric view of the natural world (Marriott, 2002; Sackes et al., 2009; Ganea et al., 2011). Picture books are a significant source of information about the biological world for young children and yet the majority of books for young children present the natural world in highly distorted ways (Marriott, 2002). Animal characters exhibit human characteristics and their natural environments are distorted, thus raising the question of how these representations of animals in picture books affect children's understanding of the biological world. Books that anthropomorphize animals may lead children to take a scientifically inaccurate view of the natural world by attributing human-like characteristics to non-human animals. This effect may be a result of seeing depictions of animals like humans (wearing clothes and engaging in human-like activities) and/or hearing descriptions of animals that include references to intentional human-like states and activities. The current research attempts to disentangle the contributing role of these two factors on children's learning and reasoning about non-human animals. More specifically, we will (1) ask whether children's ability to learn new biological information about a novel animal is affected by the type of picture book they are exposed to, and (2) determine the relative contribution that anthropomorphic images and language play in children's attributions of traits to real animals.

## Study 1

The goal of Study 1 was to examine the effect of anthropomorphic language, used to describe novel animals in picture books, on both children's learning of facts about the novel animals and on their willingness to anthropomorphize biological and psychological properties of animals. Children were exposed to books that had realistic images of novel animals, however the language used to describe them was either factual or anthropomorphic. Thus, children were assigned to one of two book conditions: *No Anthropomorphism* (realistic images and factual language) or *Anthropomorphic Language* (realistic images and anthropomorphic language).

### Methods

#### Participants

Seventy-five children were recruited in the Boston metropolitan area. The majority of children (*N* = 62) were recruited using public birth records. The remaining children (*N* = 13) were recruited at a local science museum. Five children were dropped because of lack of cooperation (*N* = 2), prior knowledge of the novel animals (*N* = 1), or because they showed a response bias (*N* = 2). To be included in the study children had to answer “No” to at least two out of eight questions that had a “No” answer. Participants were divided into groups of 3-year-olds (*N* = 24; *M* = 40.6 months; Range = 36.0–47.1 months), 4-year-olds (*N* = 24; *M* = 53.0 months; Range = 48.0–59.6 months), and 5-year-olds (*N* = 22; *M* = 66.7 months; Range = 60.1–71.9 months). Females made up 50% (*N* = 12) of the 3-year-old sample, 50% (*N* = 12) of the 4-year-old sample, and 59% (*N* = 13) of the 5-year-old sample. Most of the participants were white (72%), but the sample also included Asian (5%), Black (2%), Hispanic (3%), and Mixed Race (11%) participants. An additional 7% of families declined to disclose ethnicity information. The majority of participants came from middle class families.

#### Materials

Six picture books were specifically designed for this study, featuring three animals that are unfamiliar to most children: cavies, oxpeckers, and handfish. The animals were chosen based on pilot testing in which children were asked to identify a series of animals from photographs. The three animals used in the picture books were those that were identified correctly least often, with children in the pilot study most frequently indicating that they did not know the names for these animals.

**Figure 1:**
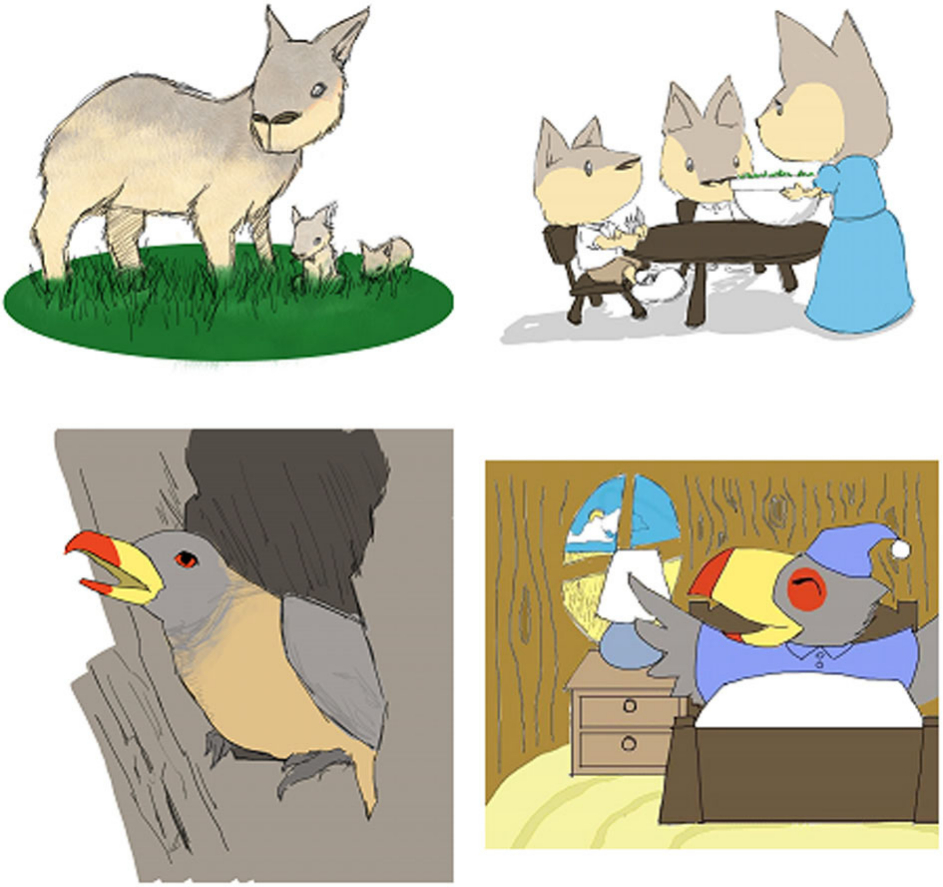
**Examples of realistic (left) and anthropomorphic (right) illustrations used in Study 1 and Study 2, respectively**.

For each animal, two books were created according to condition. Both of these books provided three facts about the animal. The facts were about where the animal lives, what the animal eats, and one other interesting fact (e.g., “Oxpeckers sit on the backs of large animals, like rhinoceros.”). In the *No Anthropomorphism* condition the book featured realistic images and factual language. In the *Anthropomorphic Language* condition, the book featured realistic images and anthropomorphic language (e.g., “Mother cavy tucks her babies into bed in a small cave… ‘Don’t be afraid,' she says. ‘I’ll listen for noises with my big ears to keep us safe.”'). Supplementary Materials contains samples of the stories used in the two conditions, and Figure 1 shows sample illustrations used in the two books; the left column images are sample images from the books used in Study 1. In addition to the picture books, color photographs of each real animal were used during the test phase of the study.

#### Design and procedure

Children were tested either in the laboratory or at the science museum. In both settings, children sat at a table to read the picture books with Experimenter 1 (E1). Experimenter 2 (E2) sat at a small side table with a large pile of papers and photographs as well as several file folders. The picture books were kept on a small shelf in a semi-hidden area, away from the main table. Children were assigned to either the *No Anthropomophism* or the *Anthropomorphic Language* book condition. Each child read one book about each of the animals with the experimenter, for a total of three books in each condition.

Each child was first greeted by E1, and spent a few minutes playing and warming up. E1 then led the child to the table, saying that they would read some books together. When E1 and the child arrived at the table, E1 introduced E2, saying, “This is my friend. She's going to do some work while we read.” E2 then confirmed E1's statement, saying, “Yes, I have to organize all of these papers and pictures! I'll just be working while you read.” E1 and the child then read the first book twice, to ensure that the child attended to the story. After they finished the book, E1 said, “I have another book we can read. You stay here and I'll go find it.”

After E1 left the table, E2 approached the child and said, “I heard you reading about the cavy (or oxpecker or handfish), and guess what? I found this picture of a *real* cavy. I don't know anything about cavies. Can you help me answer some questions about this *real* cavy?” E2 emphasized to the child that the animal in the picture was real and that she did not know anything about the animal.

E2 then proceeded to ask the child six yes/no questions about the animal. All test questions can be found in Supplementary Materials. The questions included two factual questions about information that had been presented in the books used in both conditions, and two anthropomorphic questions that had been presented as things the animals did in the *Anthropomorphic Language* condition (e.g., talk, have friends). Of the six anthropomorphic questions across the three books, two focused on physical behaviors, two asked about human emotions, and two concerned social understanding. Two factual control questions, which were not taught in the books, were also included, to ensure that children's answers to the factual questions were gained from the book, and that they were not simply using their own background knowledge about animals.

Answers to the questions were counter-balanced, so that the correct answer for three of the questions was “yes” and for three of the questions was “no.” Each child was asked the questions in the same order. After the child had answered all of the questions, E2 said, “Thanks for your help! I'm going to go back to my work. I think E1 will be back soon.” Once E2 had gone back to the side table, E1 returned with the second book, and the procedure was repeated for that book, and again for the third book. The order in which the books were read was counterbalanced across children.

Children's answers to each question were scored dichotomously online, so that they received a score of “1” for each correct answer and a score of “0” if they gave an incorrect answer. A second coder scored 54% percent of the participants (*N* = 38) from videotape. Inter-rater reliability between the two coders was high (kappa = 0.90), and all discrepancies were resolved through discussion between the coders.

### Results and discussion

We first determined the proportion of correct answers (out of six) that children provided for both the factual and control questions, and the proportion of times children did *not* extend anthropomorphic characteristics to the animals in response to the anthropomorphic questions. Thus, for anthropomorphic questions, higher scores reflect lower levels of anthropomorphizing. The means and standard deviations are provided in Table 1 for each age group. Boys and girls did not differ, on average, in their answers to any of the types of questions.

**Table 1:** **Mean proportion of correct responses for children in the No Anthropomorphism (NA) and Anthropomorphic Language (AL) conditions, Study 1**.

**Question type**	**3 year olds**		**4 year olds**		**5 year olds**	
	**NA**	**AL**	**NA**	**AL**	**NA**	**AL**
	***M* (*SD*)**	***M* (*SD*)**	***M* (*SD*)**	***M* (*SD*)**	***M* (*SD*)**	***M* (*SD*)**
Factual	0.83 (0.17)	0.75 (0.23)	0.92 (0.14)	0.96 (0.07)	0.98 (0.06)	0.94 (0.13)
Control	0.62 (0.23)	0.55 (0.17)	0.58 (0.29)	0.51 (0.28)	0.42 (0.19)	0.55 (0.22)
Anthropo-morphic	0.41 (0.30)	0.32 (0.24)	0.56 (0.30)	0.40 (0.30)	0.63 (0.18)	0.40 (0.24)

First, we asked whether the type of language used in the book (anthropomorphic vs. factual) affected children's learning of novel facts about the target animals. To answer this question we conducted a 3 (age) × 2 (condition) × 2 (question type) mixed-effects ANOVA, with age (3, 4, 5) and condition (*No Anthropomorphism, Anthropomorphic Language*) as between-subjects factors and question type (factual, control) as a within-subjects factor. This analysis revealed a significant effect of question type, *F*_(1, 64)_ = 104.68, *p* < 0.01, η*p*^2^ = 0.62, indicating that children performed significantly better on the factual questions (*M* = 0.90) than on the control questions (*M* = 0.54), *p* < 0.01. There was also a significant interaction between question type and age, *F*_(2, 64)_ = 5.00, *p* < 0.01, η*p*^2^ = 0.14. Simple effects analysis indicated a significant effect of age, *F*_(2, 64)_ = 8.84, *p* < 0.01, and Tukey pairwise comparisons showed that both 4- and 5-year-olds performed significantly better than 3-year-olds on the factual questions (*p* < 0.01, for both), but that there were no differences between the age groups on the control questions. Thus, although the children may have entered the task with some previous general knowledge about animals, the significant difference between their performances on the factual vs. the control questions indicates that children, especially the 4- and 5-year-olds, learned new facts from both types of books.

Second, we asked whether the type of language used in the book had an influence on how likely children were to attribute human-like characteristics to real animals. For this analysis we considered children's attribution of anthropomorphic characteristics across the two conditions. A 2 × 3 ANOVA examining children's answers to the anthropomorphic questions in terms of condition (*No Anthropomorphism, Anthropomorphic Language*) and age (3, 4, 5), revealed a main effect of condition, *F*_(1, 64)_ = 6.43, *p* < 0.05, η*p*^2^ = 0.09. This indicates that children in all age groups were more likely to attribute anthropomorphic characteristics to real non-human animals after hearing a story with anthropomorphic language than after hearing a story with realistic language. Although there were no significant differences across age groups, the difference in attribution of anthropomorphic characteristics between conditions was most pronounced in 5-year-olds (*Mdiff* = 0.23).

Third, we asked whether children's attribution of anthropomorphic traits to non-human animals varies with the type of characteristic: physical behaviors (e.g., “Do cavies talk?”), emotions (e.g., “Can handfish feel proud?”), and social understanding (e.g., “Do oxpeckers have friends?”). A 3 (age) × 2 (condition) × 3 (type of anthropomorphism) mixed-effects ANOVA with age (3, 4, 5) and condition (*No Anthropomorphism*, *Anthropomorphic Language*) as between-subjects factors and type of anthropomorphism (behavior, emotion, social understanding) as a within-subjects effect was carried out. Significant effects of condition, *F*_(1, 64)_ = 6.36, *p* < 0.05, η*p*^2^ = 0.09, and type of anthropomorphism, *F*_(2, 128)_ = 19.89, *p* < 0.01, η*p*^2^ = 0.237, were found, revealing that children were less likely to extend anthropomorphic characteristics to animals in the *No Anthropomorphism* condition (*M* = 0.53) than in the *Anthropomorphic Language* condition (*M* = 0.37). Further, overall, children were less likely to extend anthropomorphic physical behaviors to animals (*M* = 0.63) than they were to extend either human-like emotions (*M* = 0.36) or social understanding (*M* = 0.37). A significant interaction between type of anthropomorphism and condition, *F*_(2, 128)_ = 3.58, *p* < 0.05, η*p*^2^ = 0.07 was also found. Simple effects analysis indicated that children were more likely to extend both anthropomorphic physical behaviors, *F*_(1, 64)_ = 7.02, *p* < 0.01, and emotions, *F*_(1, 64)_ = 7.86, *p* < 0.01, in the *Anthropomorphic Language* condition than they were in the *No Anthropomorphism* condition, but there was no difference in their endorsement of anthropomorphic social understanding. This reveals that, like adults, young children seem to have a less clear conception of differences between humans and other animals in regard to mental characteristics, as opposed to behaviors (Horowitz and Bekoff, 2007). However, exposure to anthropomorphized language may encourage them to attribute more human-like characteristics to other animals than exposure to factual language.

To summarize, the results of Study 1 indicate that preschoolers can learn simple facts about animals from books, whether the information is presented to them in a context that uses realistic or anthropomorphic language to describe the animals. This ability is more robust in 4- and 5-year-olds than in 3-year-olds. This finding is consistent with the results of Ganea et al. (2011) regarding the learning of simple biological information (e.g., color camouflage) from picture books that varied the type of language (realistic vs. intentional) used.

The results also show that the type of language used in books affects how likely children are to attribute anthropomorphic traits to real animals. Children were more likely to say that real animals feel human emotions or even talk after listening to stories that used anthropomorphic rather than realistic language. There are two ways to explain this effect: either that the anthropomorphic language increases children's tendency to attribute anthropomorphic traits to animals, or that hearing realistic language suppresses their natural inclination to attribute human-like traits to other non-human animals. This question is examined in greater detail in Study 2.

## Study 2

The results of Study 1 showed that the language children hear in picture books has important implications for their attribution of anthropomorphic traits to animals in the real world, though not necessarily for their learning of factual information. In Study 2, we aimed to: (1) examine the contributing role of images, alone or in combination with language, on children's learning and reasoning about novel animals, and (2) ascertain whether anthropomorphism in picture books increases children's willingness to endorse anthropomorphic traits in animals, or whether realistic books serve to decrease children's natural anthropomorphic tendencies.

To address the first goal, children were exposed to books that had unrealistic images of novel animals, and the language used to describe them was either factual or anthropomorphic. Thus, children were assigned to one of two book conditions: *Anthropomorphic Pictures* (anthropomorphic images and factual language) or *Full Anthropomorphism* (anthropomorphic images and anthropomorphic language). In this study, parents were also asked to complete a questionnaire that asked about the average amount of time they spent reading books with their children each week, and the types of books their children enjoyed reading.

To address the second goal, a control *No Book* condition was run in which 5-year-old children were shown the photographs of the target real animals and asked the test questions without prior exposure to a picture book. Children's performance in this condition would provide an estimated baseline for 5-year-olds' tendency to attribute anthropomorphic characteristics to non-human animals, and thus, could be compared to their performance in the other conditions in this study. If 5-year-olds were just as likely to extend anthropomorphic characteristics in this condition as they were in the *No Anthropomorphism* condition in Study 1, than the difference seen in the *Anthropomorphism* conditions may reflect an enhancing effect of anthropomorphic picture books on children's anthropocentric reasoning.

### Methods

#### Participants

Eighty-eight children were recruited from the Greater Toronto Area. Children were recruited from an existing database of families who provided their contact information at local family-oriented festivals and infancy and childhood fairs. Sixteen children were excluded from the final analysis due to inattentiveness during testing (*N* = 2), a “yes” response bias (*N* = 13), or because they fell outside of the age range at the time of testing (*N* = 1). Participants were divided into two groups: 3-year-olds (*N* = 27; *M* = 43.8; Range = 36.4–47.9 months) and 5-year-olds (*N* = 45; *M* = 66.10; Range = 60.8–71.7). Because Study 1 indicated no overall differences between 4-year-olds and 5-year-olds, Study 2 focused exclusively on 3- and 5-year-olds. Females made up approximately half of the 5-year-olds (*N* = 13), and 63% (*N* = 17) of the 3-year-olds. As in Study 1, the majority of the participants were white (60%). The sample also included Black (2%), Asian (4%), and Mixed Race (8%) participants. An additional 11% of participants identified themselves as “Other,” and ethnicity information was not provided by 15% of the participants. The majority of participants came from middle class families.

#### Materials

Six picture books were created by the experimenters, featuring the same novel animals that were used in Study 1: oxpeckers, cavies, and handfish. In this set of books, we manipulated the illustrations by featuring anthropomorphized depictions of the animals in all six books (see Figure 1, sample images on the right side column). As in Study 1, a photograph of each real animal was used during the test phase. The type of language used to describe the animals varied as a function of condition, either anthropomorphic or factual.

#### Design and procedure

All children were tested in a laboratory setting. The procedure was the same as that used in Study 1, except that parents were also given the questionnaire on book reading behavior mentioned above. Twenty-seven children from each age group were randomly assigned to either an *Anthropomorphic Pictures* condition or a *Full Anthropomorphism* condition. An additional 18 5-year-olds were assigned to the control *No Book* condition. Only 5-year-olds were assigned to this condition because the type of picture book used seemed to have the greatest effect on these older preschoolers in Study 1. Similarly to Study 1, children's answers to all questions were scored dichotomously online. A second coder scored 61% of the participants (*N* = 33) from videotapes. Interrater agreement was high, kappa = 0.97, and disagreements were resolved through discussion between the coders.

### Results and discussion

The analyses were the same as in Study 1. Although girls made up slightly more than half of the 5-year-old sample, there were no differences between boys and girls on any of the test questions. Means and standard deviations for each age group are provided in Table 2.

**Table 2:** **Mean proportion of correct responses for children in the Anthropomorphic Pictures (AP), Full Anthropomorphism (FA), and No Book control condition in Study 2**.

**Question type**	**3 year olds**		**5 year olds**		
	**AP**	**FA**	**AP**	**FA**	**Control**
	***M* (*SD*)**	***M* (*SD*)**	***M* (*SD*)**	***M* (*SD*)**	***M* (*SD*)**
Factual	0.90 (0.11)	0.75 (0.21)	0.93 (0.15)	0.89 (0.15)	0.68 (0.21)
Control	0.45 (0.30)	0.52 (0.27)	0.55 (0.32)	0.63 (0.23)	0.64 (0.24)
Anthropomorphic	0.54 (0.24)	0.38 (0.29)	0.56 (0.23)	0.29 (0.19)	0.50 (0.23)

First, we analyzed children's performance on the factual vs. control questions as a function of book condition. A 2 (age) × 2 (condition) × 2 (question type) mixed-effects ANOVA, with age (3, 5) and condition (*Anthropomorphic Pictures*, *Full Anthropomorphism*) as between-subjects factors, and question type (factual, control) as a within-subjects factor, indicated a significant effect of question type, *F*_(1, 50)_ = 61.99, *p* < 0.01, η*p*^2^ = 0.55, a significant effect of age, *F*_(1, 50)_ = 4.29, *p* < 0.05, η*p*^2^ = 0.08, and a significant interaction between question type and condition, *F*_(1, 50)_ = 4.20, *p* < 0.05, η*p*^2^ = 0.08. As in Study 1, overall, children performed significantly better on factual questions (*M* = 0.87) than on control questions (*M* = 0.54), and 5-year-olds (*M* = 0.75) performed significantly better than 3-year-olds (*M* = 0.65). Further, simple effects analysis indicated that children answered fewer factual questions correctly in the *Full Anthropomorphism* condition than in the *Anthropomorphic Pictures* condition, *F*_(1, 50)_ = 4.99, *p* < 0.05. There was no significant difference between conditions on children's performance on the control questions. These results reveal that, although children were able to learn factual information about the animals from the picture books, they were less likely to do so when the books contained both anthropomorphic pictures and language.

Second, to examine the effect of the type of book on children's attribution of anthropomorphic traits, a 2 × 2 ANOVA was used, examining children's answers to the anthropomorphic questions in terms of condition (*Anthropomorphic pictures*, *Full Anthropomorphism*) and age (3, 5). A main effect of condition was found, *F*_(1, 50)_ = 10.26, *p* < 0.01, η*p*^2^ = 0.17, indicating that children's scores on the anthropomorphic questions were significantly higher in the *Anthropomorphic Pictures* condition than in the *Full Anthropomorphism* condition. Thus, children who were exposed to books containing both anthropomorphic images and language were more likely to extend anthropomorphic characteristics to real animals than were children exposed to books with anthropomorphic images and factual language.

Third, as in Study 1, we asked whether children's endorsement of anthropomorphic characteristics differed according to the type of characteristic about which they were asked. A 2 × 2 × 3 ANOVA with age group (3, 5) and condition (*No Anthropomorphism*, *Anthropomorphic Language*) as between-subjects factors and type of anthropomorphism (behavior, emotion, social understanding) as a within-subjects effect revealed a significant effect of type of anthropomorphism *F*_(2, 100)_ = 17.89, *p* < 0.01, η*p*^2^ = 0.26, and a significant effect of condition *F*_(1, 50)_ = 10.23, *p* < 0.01, η*p*^2^ = 0.17. Overall, children were less likely to endorse anthropomorphic physical behaviors (*M* = 0.65) than they were to endorse anthropomorphic emotions (*M* = 0.33) or social understanding (*M* = 0.35). Children were also less likely to extend anthropomorphic traits to animals in the *Anthropomorphic Pictures* condition (*M* = 0.55) than they were in the *Full Anthropomorphism* condition (*M* = 0.33). Significant interactions between type of anthropomorphism and age, *F*_(2, 100)_ = 5.72, *p* < 0.01, η*p*^2^ = 0.10, as well as condition, *F*_(2, 100)_ = 4.84, *p* < 0.05, η*p*^2^ = 0.09 were also found. Simple effects analyses indicated that children were more likely to extend both anthropomorphic behaviors, *F*_(1, 50)_ = 12.37, *p* < 0.01, and emotions, *F*_(1, 50)_ = 11.63, *p* < 0.01 to real animals in the *Full Anthropomorphism* condition than in the *Anthropomorphic Pictures* condition. There was no difference in children's attribution of social understanding to animals between conditions, but there was a significant effect of age, *F*_(1, 50)_ = 6.66, *p* < 0.05. Tukey pairwise comparisons indicated that, in the *Anthropomorphic Pictures* condition, 5-year-olds were significantly less likely to endorse anthropomorphic behaviors than they were to endorse emotions or social understanding (*p* < 0.01, for both).

To summarize, the results of Study 2 indicate that anthropomorphic illustrations in and of themselves have little effect on the information children take away from picture books. Both 3-year-olds and 5-year-olds learned factual information from books with anthropomorphic pictures. However, when anthropomorphic language and pictures were combined (*Full Anthropomorphism*), children at both ages were less likely to learn the facts from the books and apply them to the real animal.

A similar effect is seen in the effect of anthropomorphic illustrations on children's conceptions of animals' traits. Children were more likely to say that real animals have human-like characteristics after listening to stories that used *both* anthropomorphic language and images, than after listening to stories that used anthropomorphic images and realistic language. A comparable result was seen in Study 1 when realistic illustrations were used. In that study, children were more likely to attribute anthropomorphic characteristics to non-human animals after hearing stories containing anthropomorphic language. Taken together, these results indicate that the language in children's picture books may be more important than illustrations in children's conceptions of animals as human-like, but that the combination of both anthropomorphic language and pictures may make learning of facts from picture books difficult even for older preschoolers.

#### How anthropomorphic books affect anthropomorphic reasoning

**Figure 2:**
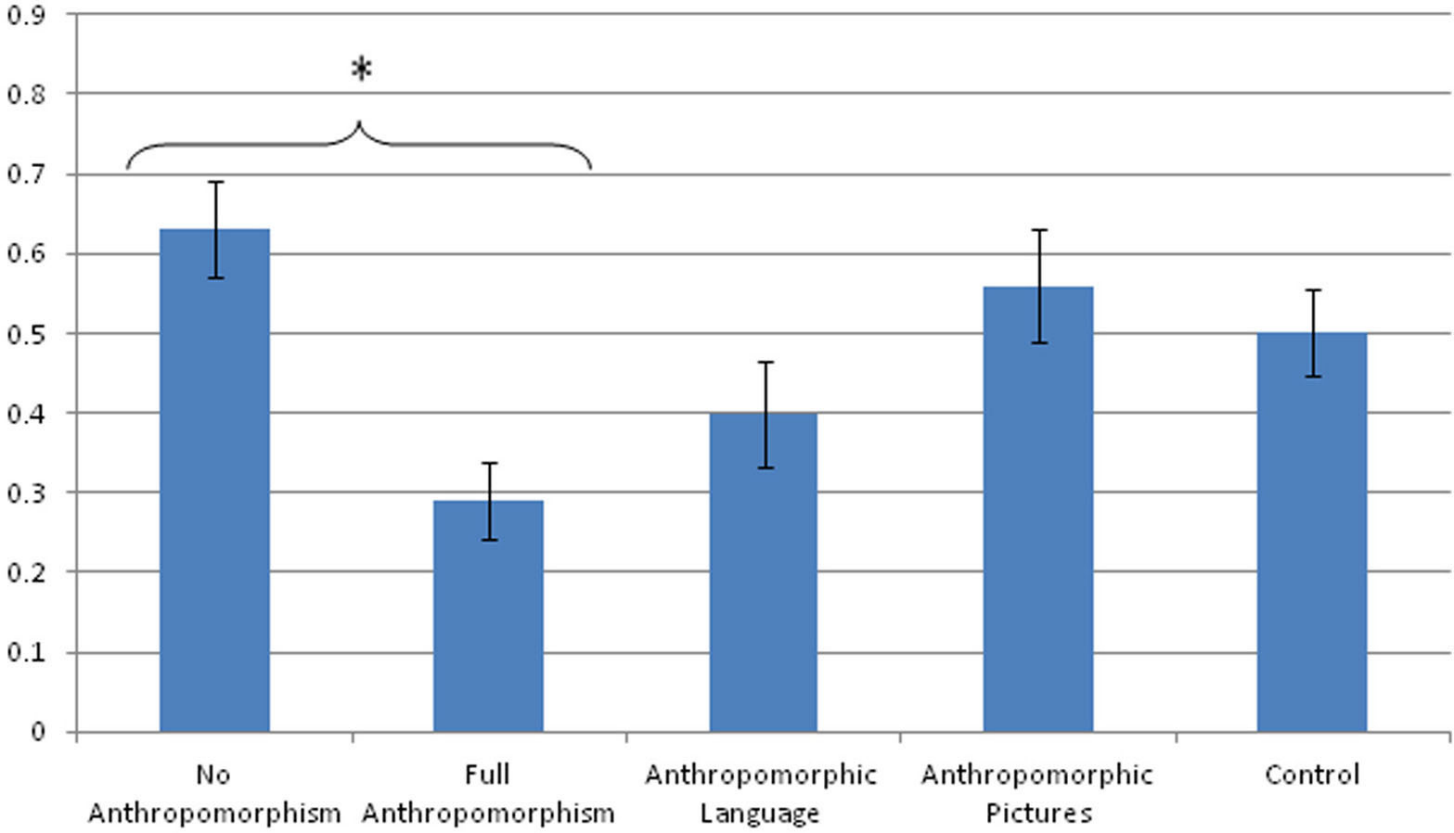
**Mean proportions of 5-year-olds' correct responses to the anthropomorphic questions across all conditions**. ^*^p < 0.05.

The analyses in Studies 1 and 2 examining the effect of book type on how likely children were to attribute anthropomorphic traits to animals could not specify whether the anthropomorphic books enhanced children's extension of anthropomorphic traits to real animals, or whether realistic books inhibited children's tendency to anthropomorphize more generally. To be able to answer this question we compared children's performance in the conditions so far to a baseline control condition in which children were not read a storybook. This control condition was administered only to 5-year-olds, because the level of anthropomorphism in 3-year-olds did not vary much as a function of condition. A One-Way ANOVA comparing 5-year-olds' attribution of anthropomorphic traits across all conditions was performed. There was a significant effect of condition, *F*_(4, 66)_ = 4.52, *p* < 0.01, η*p*^2^ = 0.13, and Tukey pairwise comparisons indicated that this effect was driven by the difference between the *No Anthropomorphism* and *Full Anthropomorphism* conditions (*p* < 0.01). Children were more likely to attribute human-like characteristics to real animals when exposed to a book with both anthropomorphic language and pictures than when exposed to a book containing realistic language and illustrations (Figure 2). There was a trend approaching significance for children in the *No Book* control condition to attribute fewer anthropomorphic traits to animals than did the children in the *Full Anthropomorphism* condition (*p* = 0.07).

Taken together, these findings indicate that anthropomorphic picture books, and especially books that combine anthropomorphic pictures and language, seem to encourage young children to endorse anthropomorphic traits for non-human animals. After reading such books, children were more likely to attribute human traits to real animals, and this endorsement of anthropomorphic characteristics was stronger than children's baseline anthropomorphism.

#### Possible predictors of children's learning from picture books

We also asked whether children's performance in this study was influenced by their general exposure to picture books or other demographic variables. Based on parents' responses to questionnaires, there were no differences between age groups, conditions, or genders in children's ethnicity or in SES variables, including parents' education and employment. Further, there were no mean differences between children in each condition or in each age group in terms of the amount of time they spent engaged with books each week, or their overall preferences for different types of books (fantasy vs. realistic books about animals; books that have photographs vs. drawings).

There were, however, some differences in book preferences between genders. For both 3- and 5-year-olds, parents reported that boys liked books about vehicles [*t*_(25)_ = −5.13, *p* < 0.01, *d* = −2.05, *t*_(25)_ = −2.79, *p* < 0.05, *d* = −1.12] and books about how things work [*t*_(25)_ = −2.68, *p* < 0.05, *d* = −1.07, *t*_(25)_ = −2.54, *p* < 0.05, *d* = −1.02] more than girls did. There were no other gender differences in children's preferences for different types of books. These preferences were unrelated to children's performance on the experimental task.

A multiple regression analysis indicated that neither children's SES, enjoyment of picture books, the amount of time spent reading each week, nor the specific types of books that children preferred to read were predictive of children's performance on the factual, control, or anthropomorphic questions.

## General discussion

Many books for young children present animals in fantastical and unrealistic ways, as wearing clothes, talking and engaging in human-like activities. The research presented here suggests that books that anthropomorphize animals can affect children's conceptions of animals. Overall children in this research were less likely to attribute anthropomorphic characteristics to animals when exposed to books that presented animals in a realistic rather than anthropomorphic manner. Our results are consistent with recent findings indicating that 5-year-olds were less likely to transfer a new biological property from one non-human animal to others after reading a book that presented bears from an anthropomorphic perspective rather than from a biological perspective (Waxman et al., 2014). The current research expands on this finding by disentangling the contributing role of images and language in picture books on children's learning and reasoning about animals.

More specifically, after hearing stories that used anthropomorphic language to describe unfamiliar animals, preschoolers in the current research were more likely to extend both human physical behaviors and emotions when asked questions about those real animals. This effect was present even in a book condition where only the language was anthropomorphized (Study 1). Thus, even when seeing realistic images of the animals and their environment, if the language used to describe them was animistic, children were more likely to attribute anthropomorphic traits when asked questions about the real animals compared to children who heard realistic language. Although this tendency did not differ as a function of age, it seemed stronger in the 5-year-olds in this research. There is evidence that anthropomorphism may be acquired between 3 and 5 years of age in urban children (Herrmann et al., 2010) and thus it is possible that the younger children may not have yet developed the same level of sensitivity to typical cultural input (language in particular) about biological phenomena as the 5-year-olds.

A second important finding from this research is that children learned more facts about animals from books that used factual language and/or realistic illustrations to describe the animals. When children in Study 2 were exposed to books where anthropomorphic images and language were combined they were less likely to apply the facts to photographs of the real animals compared to a book that used only anthropomorphic images. This type of book, which combines both fantastical language and anthropomorphic illustrations of animals, is typical of commercially available books. Our results suggest that this combination may create a story context that is too dissimilar from reality for preschoolers to realize that information important for the real world is being conveyed. As children get older and have more experience with fantastical stories, they may acquire knowledge that information encountered in fantastical books can be relevant to the real world, but the current findings indicate that this is not yet the case for preschool-aged children. This effect is especially true when both the images and language used in the story were fantastical—children learned fewer facts about real animals in this condition.

This research adds to the growing body of literature on how picture-book features support or detract from young children's learning and holds important implications for a wide audience. These findings inform parents and teachers about how to select pictures books that will aid children's transfer of factual information from books to the real world. Together with related research cautioning on the use of fantasy features in educational books (Richert et al., 2009; Ganea et al., 2011; Walker et al., 2012a), this work suggests that if the goal of the picture book interaction is to teach children information about the world, it is best to use books that depict the world in a realistic rather than fantastical manner. More specifically, if we want children to learn new things about animals, we need to expose them to stories that present the animals and their environments in a biologically realistic manner, both in the way that they are depicted and the way they are described. Based on these results, teachers might also choose to supplement their picture book selection in the classroom with live display of animals to aid children's biological conceptions of real animals and their habitats.

Recent findings with adults show that there are individual differences in adults' tendency to anthropomorphize nature (Waytz et al., 2010). Developmental research examining children's anthropomorphism has also shown that the tendency to attribute a property to an animal once it was introduced for a human base is not as prevalent at 3 years of age as it is at 5 years of age (Herrmann et al., 2010) and it is most common in urban cultures (Ross et al., 2003; Waxman and Medin, 2007). Our research suggests that an important factor in the development of anthropomorphism in childhood may be exposure to media (e.g., picture books, television) that commonly portrays animals and other inanimate entities with human-like characteristics (see also Waxman et al., 2014). Such portrayals can lead children to think of entities in the natural world as imbued with intentions and human-like states.

As cultural artifacts, books can communicate a culture's epistemological orientation toward nature (Dehghani et al., 2013), and thus, they can provide opportunities to introduce children to science concepts early on. The tendency to reason about the biological world from an anthropocentric point of view can have negative consequences for children's causal biological understanding. For example, research with older children has shown they have more difficulty accurately interpreting evolutionary change when those concepts are presented using anthropomorphic language (Legare et al., 2013). In light of the current findings and the documented potential of books to introduce young children to concepts about natural phenomena early on (Ganea et al., 2011; Kelemen et al., 2014), future research should further investigate the ways in which picture books can influence children's reasoning about the natural world. In the current research we examined preschoolers' reasoning in relation to the specific animals presented in the books they were exposed to. The results show that children are more likely to endorse anthropocentric traits for specific animals after being exposed to books that anthropomorphize those animals than after being exposed to books that present the animals in a realistic manner. Future research should examine whether the type of books children read will also affect how likely children are to endorse anthropomorphic traits to other novel animals that they have not read about. It would also be important to know whether books have a similar impact on children growing in different communities. For example, we might predict that anthropomorphic books might have less impact on children who have more direct contact with animals and who are generally exposed to discourse about animals that is not human-centered.

To conclude, picture books are an important source of information about the world and in particular about things and events that children cannot experience directly. Despite their potential to broaden children's general knowledge about the world, only a very small percentage of the books teachers select for their classrooms are informational and non-fiction books, both in preschool (Yopp and Yopp, 2006; Pentimonti et al., 2011) and first grade (Duke, 2000). Most of the books in classrooms for young children fall along a continuum from purely fictional storybooks to hybrid books that include a mixture of fantasy and factual information in a narrative format. Although these types of books are important for other aspects of children's development, the research presented here points to the importance of carefully considering the type of books that we use with young children when teaching them new information about the world. Books that do not present animals and their environments accurately from a biological perspective may not only lead to less learning but also influence children to adopt a human-centered view of the natural world.

## Conflict of interest statement

The authors declare that the research was conducted in the absence of any commercial or financial relationships that could be construed as a potential conflict of interest.
