# Supplementary material for: Do cavies talk? The effect of anthropomorphic picture books on children's knowledge about animals
Source: Front Psychol. 2014 Apr 10;5:283. doi: 10.3389/fpsyg.2014.00283 (PMC3989584; doi:10.3389/fpsyg.2014.00283)
Supplement: Supplementary file 1 [file Presentation1.PDF]

Appendix A

Sample Stories

Realistic

When the mother cavy wakes up, she usually eats lots of grass and other plants.

Then the mother cavy feeds her baby cavies.

Mother cavy also licks the babies' fur to keep them clean.

Mother cavy and her babies spend the rest of the day lying in the sun.

At night, they sleep in a small cave.

After they go to sleep, mother cavy's big ears help her hear noises around her.

Fantasy

"Yum, these grass and plants are delicious!" Mother cavy thinks as she eats her breakfast.

"I will feed some to my baby cavies too!" she says.

The baby cavies love to play in the grass! But they've gotten all dirty! "Time for your bath," Mother cavy says.

Mother cavy and her babies like to spend the afternoon sunbathing.

At night, Mother cavy tucks her babies in to bed in a small cave. "Mom, I'm scared!" says the baby cavy.

"Don't be afraid," she says. "I'll listen for noises with my big ears and keep us safe."

Appendix B

Test Questions

Handfish

Do handfish eat worms (factual)? Yes

Do handfish get excited (anthropomorphic)? No

Do handfish lay eggs (control)? Yes

Do handfish live in shallow water (control)? No

Do handfish move slowly (factual)? Yes

Can handfish be proud (anthropomorphic)? No

Oxpecker

Do oxpeckers live under bushes on the ground (factual)? No

Do oxpeckers fly (control)? Yes

Can oxpeckers have friends (anthropomorphic)? No

Do oxpeckers make a lot of noise (control)? Yes

Do oxpeckers worry/care about rhinos (anthropomorphic)? No

Do oxpeckers eat ticks (factual)? Yes

Cavy

Do cavies eat grass (factual)? Yes

Do cavies talk (anthropomorphic)? No

Are cavies good diggers (control)? Yes

Do cavies sleep in trees (factual)? No

Can cavies jump high (control)? Yes

Do baby cavies take baths (anthropomorphic)? No
